# LncRNAs Ride the Storm of Epigenetic Marks

**DOI:** 10.3390/genes16030313

**Published:** 2025-03-06

**Authors:** Giulia Gaggi, Clinton Hausman, Soomin Cho, Brianna C. Badalamenti, Bon Q. Trinh, Annalisa Di Ruscio, Simone Ummarino

**Affiliations:** 1 Department of Medicine and Aging Sciences, “G. D’Annunzio” University of Chieti-Pescara, 66100 Chieti, Italy; giulia.gaggi@unich.it; 2 UdA-TechLab, “G. D’Annunzio” University of Chieti-Pescara, 66100 Chieti, Italy; 3 Harvard Stem Cell Institute, Harvard Medical School, Boston, MA 02115, USA; chausman@bidmc.harvard.edu (C.H.); scho4@bidmc.harvard.edu (S.C.); bcbada26@bu.edu (B.C.B.); 4 Beth Israel Deaconess Medical Center, Cancer Research Institute, Boston, MA 02215, USA; 5 Harvard Medical School Initiative for RNA Medicine, Harvard Medical School, Boston, MA 02115, USA; 6 Department of Pathology, University of Virginia School of Medicine, Charlottesville, VA 22908, USA; mbj5ez@virginia.edu; 7 Molecular Genetics & Epigenetics Program, University of Virginia Comprehensive Cancer Center, Charlottesville, VA 22908, USA; 8 Department of Biology, Tufts University, Medford, MA 02155, USA

**Keywords:** ncRNA, epigenetics, epigenetic marks, chromatin, epigenetic memory

## Abstract

Advancements in genome sequencing technologies have uncovered the multifaceted roles of long non-coding RNAs (lncRNAs) in human cells. Recent discoveries have identified lncRNAs as major players in gene regulatory pathways, highlighting their pivotal role in human cell growth and development. Their dysregulation is implicated in the onset of genetic disorders and age-related diseases, including cancer. Specifically, they have been found to orchestrate molecular mechanisms impacting epigenetics, including DNA methylation and hydroxymethylation, histone modifications, and chromatin remodeling, thereby significantly influencing gene expression. This review provides an overview of the current knowledge on lncRNA-mediated epigenetic regulation of gene expression, emphasizing the biomedical implications of lncRNAs in the development of different types of cancers and genetic diseases.

## 1. Introduction

**Figure 1:**
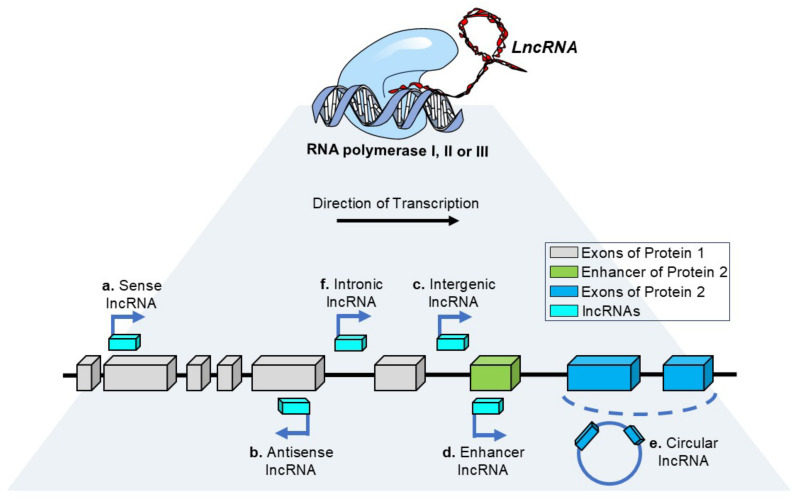
**Classification of long non-coding RNAs according to their transcriptional properties.** Long non-coding RNAs are divided into six distinct subtypes based on their transcriptional characteristics, which include the following: (**a**) sense lncRNAs, transcribed in the same direction as the adjacent protein-coding genes; (**b**) antisense lncRNAs, transcribed in the opposite direction; (**c**) intergenic lncRNAs, transcribed from regions between two protein-coding genes; (**d**) enhancer lncRNAs, transcribed from enhancer regions; (**e**) circular lncRNAs, generated through a splicing mechanism that forms circular RNA structures; and (**f**) intronic lncRNAs, transcribed from introns of protein-coding genes.

The central dogma of molecular biology states that genetic information is carried in one direction, from DNA to RNA to protein. However, only 3% of the RNAs transcribed from DNA are then translated into proteins. The rest of the RNAs are classified as non-coding RNAs (ncRNAs), which participate in regulating gene expression through different molecular mechanisms. Some non-coding RNAs (ncRNAs), like protein-coding mRNAs, are transcribed by RNA polymerase II and undergo 5′ capping and 3′ polyadenylation [1,2,3,4]. However, ncRNA genes can be transcribed by RNA polymerases I, II, or III [5,6,7,8]. Remarkably, these transcripts lack an open reading frame (ORF), which precludes their translation into proteins [9,10,11]. Based on their length, ncRNAs can be classified into additional subcategories. Long non-coding RNAs (lncRNAs) are ncRNAs longer than 200 nucleotides characterized by unique and specific expression patterns among tissues and low sequence conservation between species. lncRNAs regulate gene expression depending on their subcellular localization, whether in the nucleus or cytoplasm, and can also be categorized based on their genomic origin and orientation, including sense lncRNAs, antisense lncRNAs, intergenic lncRNAs, enhancer-associated lncRNAs, circular lncRNAs, and intronic lncRNAs [12] (Figure 1). They also arise from “pseudogenes,” which are abundant in metazoan genomes [13]; nearly 10,000 have been identified in the mouse genome [14] and almost 15,000 in the human genome [12,13,14,15]. An extended subclassification accounts for their specific roles in various molecular mechanisms, e.g., signal lncRNA, decoy lncRNA, guide lncRNA, scaffold lncRNA, and enhancer lncRNA [2,3,16,17].

In the past years, the research interest on ncRNAs has progressively increased, focusing on the role and mechanisms of lncRNAs in different diseases.

Here, we review and discuss recent insights into how lncRNAs regulate gene expression at the epigenetic level and their impact on the onset or progression of various diseases and cancers.

## 2. LncRNA Interaction with the Epigenetic Machinery

**Figure 2:**
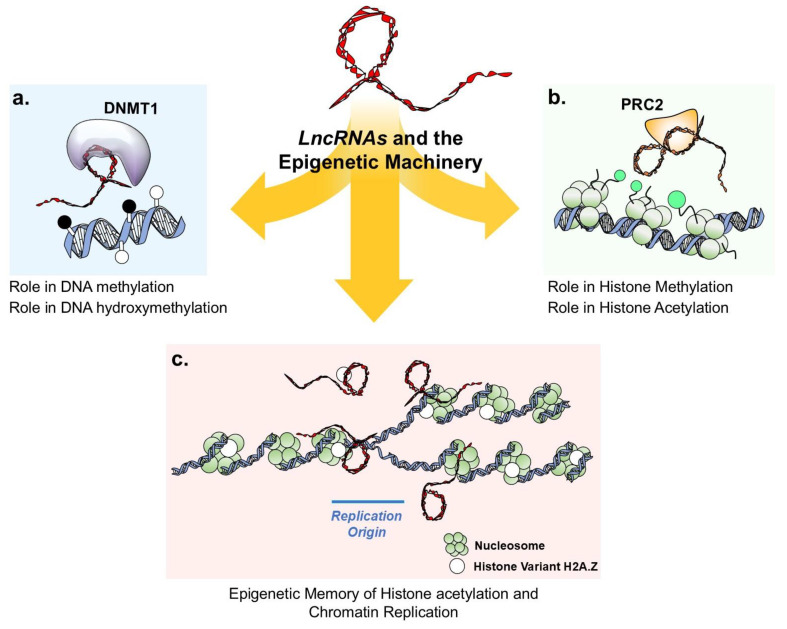
**LncRNA-mediated modulation of epigenetic machinery.** The schematic illustrates the role of Long non-coding RNAs as pivotal regulators of gene expression through their interactions with epigenetic machinery components, including (**a**) DNA methylation and hydroxymethylation enzymes. Methylated and unmethylated CpG sites are depicted by black and white dots, respectively. DNMT1 (in purple) is shown in interaction with DiR (DNMT1-interacting RNA) and provides an example of lncRN-mediated DNA methylation; (**b**) histone modifier enzymes and chromatin remodeling complexes. Green dots indicate methylated histones, PRC2 (in orange) is depicted in interaction with *HOTAIR* and provides an example of lncRNA-mediated histone methylation; (**c**) depicts the emerging hypothesis suggesting that lncRNAs play a critical role in the precise recycling and deposition of histones, such as H2A.Z, onto the nascent DNA strand during genome duplication.

LncRNA have emerged as important players in the regulation of gene expression and cellular processes. They can interact with various components of the epigenetic machinery, including DNA methylation and hydroxymethylation, histone modifications, and chromatin remodeling complexes. These interactions allow lncRNAs to participate in the modulation of gene expression programs and epigenetic states, highlighting the complexity and versatility of lncRNA-mediated gene regulation and its impact on cellular functions and disease states [18,19]. In the following paragraphs, we provide an overview of how lncRNAs interact with enzymes involved in DNA methylation, DNA hydroxymethylation, and histone acetylation, highlighting their role in maintaining epigenetic memory. A summary of these interactions is depicted in Figure 2.

### 2.1. Role of lncRNA in DNA Methylation

DNA methylation is an epigenetic mechanism that regulates gene expression. It involves the formation of 5-methylcytosine (5mC) by the DNA methyltransferase (DNMT) family proteins through the addition of a methyl group at the carbon 5 of a cytosine (C) in the context of CpG dinucleotides. The DNA methylation of promoter regions is normally associated with gene silencing [20]. In contrast, the first intron DNA methylation is associated with gene activation [21,22]. The DNMT family proteins include DNMT1, which maintains the DNA methylation status during DNA replication, and DNMT3a/3b, which are involved in the establishment of *de novo* DNA methylation patterns [2]. Despite the knowledge about the role of DNMTs and the importance of DNA methylation in regulating many cellular functions, little is still known about how sequence-specific DNA methylation is orchestrated, as DNMTs lack domains recognizing specific DNA sequences [23,24]. The discovery of a specific class of lncRNAs capable of interacting with DNMTs, DNMT-interacting RNAs (DiRs), has provided clues on the mechanism regulating the locus specificity of DNA methylation by DNMTs [23,24].

The extra-coding *CEBPA* (*ecCEBPA*) is the first DiR discovered. It arises from the same genomic locus of CCAAT Enhancer Binding Protein Alpha *(CEBPA*), whose expression is strongly regulated by DNA methylation. Di Ruscio et al. reported that the upregulation of *ecCEBPA* resulted in decrease in DNA methylation levels in the promoter region of the *CEBPA* gene and consequently in the upregulation of the *CEBPA* transcript. Interestingly, other genomic loci showed only little changes upon *ecCEBPA* perturbation, suggesting that the regulation of DNA methylation by *ecCEBPA* is locus-specific. The authors showed that *ecCEBPA* interacts with DNMT1, halting its catalytic activity and establishing a functional link between *ecCEBPA* and *CEBPA* expression [23]. Another DiR has been discovered by Chalei et al. in a mouse model. They reported that the lncRNA *Dali*, which is expressed in the central nervous system, plays a pivotal role in neuronal differentiation and can regulate the expression of many genes, affecting their DNA methylation profile by a physical bond to DNMT1. Indeed, the authors reported that the promoter region of DLG Associated Protein 5 (*DLGAP5*), High Mobility Group Box 2 (*HMGB2*), and Nitric Oxide Synthase 1 (*NOS1*) displayed an increase in DNA methylation level when *Dali* is stably knocked down, suggesting that it is able to regulate DNA methylation in a specific manner [25]. LncRNAs interacting with DNMT3a and DNMT3b, thereby contributing to the modulation of *de novo* methylation, have also been reported. For instance, Wang et al. have identified a lncRNA called *Dum* located downstream the Developmental Pluripotency Associated 2 (*DPPA2*) gene locus in a murine model and transcribed it from the opposite strand. *Dum* is induced by Myoblast determination protein 1 (MyoD) and it is involved in the differentiation of skeletal myoblast by recruiting DNMT1, DNMT3a, and DNMT3b on the *DPPA2* promoter region and silencing *DPPA2* mRNA expression [26].

More recently, Savell et al. discovered a non-coding RNA called Fos extra-coding RNA (*Fos* ecRNA) that arises from the same locus of *Fos* gene. The authors reported that incubating neurons with potassium chloride (KCl) induces the expression of both *Fos* mRNA and *Fos* ecRNA, albeit with distinct kinetics. Indeed, *Fos* ecRNA increased after 30 min upon KCl stimulation, whereas *Fos* mRNA was not significantly elevated until 1–4 h after stimulation. Based on this initial evidence, the authors emphasize the role of *Fos* ecRNA in regulating DNA methylation at the *Fos* genomic locus by interacting with DNMTs (DNMT1 and DNMT3A) in neuronal cultures. This hypothesis was subsequently validated through RNA immunoprecipitation (RIP) analyses, which demonstrated that *Fos* ecRNA physically interacts with DNMT1 and DNMT3A, leading to reduced DNA methylation in the Fos promoter region [27].

Interestingly, Merry et al. discovered 148 lncRNAs associated with DNMT1 in human colon cancer by RIP-sequencing analysis. The authors focused on the lncRNA *DACOR1*, highly expressed in normal cells and repressed in colon cancer cell lines. They showed that *DACOR1* overexpression results in an increase in DNA methylation in many gene regulatory regions involved in the control of cell metabolism and the TGF-β/BMP signaling pathway [28].

The emerging number of ncRNAs associated with intellectual disabilities have offered valuable insights into the potential role of lncRNAs in genetic disorders caused by abnormal DNA methylation, such as the *FMR1* gene in Fragile X Syndrome (FXS) [29,30,31,32]. In this particular case, FMR1-Antisense RNA 1 (Fragile X Mental Retardation 1 Antisense RNA 1, *FMR1-AS1*) seems to be involved in silencing of *FMR1*, especially in the context of Fragile X syndrome, wherein the expansion of CGG repeats leads to DNA methylation and gene silencing. While the exact mechanism remains unclear, it is hypothesized that *FMR1-AS1* may regulate the expression of *FMR1* [33]. As an antisense RNA, *FMR1-AS1* may directly interact with the *FMR1* promoter region and affect its methylation status. This interaction could either facilitate or enhance the recruitment of methylation machinery, leading to abnormal DNA methylation and gene silencing in individuals with FXS [33]. A summary of findings linking LncRNA to DNMTs is provided in Table 1.

### 2.2. Role of lncRNA in DNA Hydroxymethylation

DNA hydroxymethylation is an epigenetic modification by which a hydroxyl group is added to the C5 position of a 5-methylcytosine (5mC), generating 5-hydroxymethylcytosine (5hmC) [34]. This reaction is catalyzed by the Tet-Eleven Translocation (TET) family proteins and it is the first step in the active DNA demethylation process [35].

The TET family members include TET1, TET2, and TET3. Although it has been reported that the TET proteins have redundant activities, they have a different expression during development and in adult tissues [36]. Studies on animal models reported that TET3 is highly expressed in oocytes and zygotes but then rapidly disappears. On the contrary, TET1 and TET2 increase during preimplantation and they are highly expressed in the inner cell mass of blastocyst. In addition, TET2 is abundantly expressed in hematopoietic cells [37,38]. All TET proteins have a catalytic domain (DSBH), whereas only TET1 and TET3 show a DNA binding domain [25,28], suggesting that TET2 needs to be recruited to the DNA by other DNA binding factors, including SMAD nuclear interacting protein 1 (SNIP1), Krüppel-like factor 4 (KLF4), Purine Rich Box-1 (PU.1), and NANOG [39,40,41,42] or non-coding RNAs [43,44]

After the formation of 5hmC, TET family proteins can perform an additional oxidation step, generating 5 formylcytosine (5fC) and then 5 carboxycytosine (5caC), which are recognized and excised by a base excision repair mechanism (BER), including thymine DNA glycosylase (TDG), and replaced with an unmethylated cytosine [36]. Unlike DNA methylation, the presence of 5hmC in the gene regulatory regions is generally associated with gene expression [45,46].

It has been reported that TET2 can be recruited by ncRNAs on specific genomic loci, altering their hydroxymethylation profile. In an earlier work, Arab et al. discovered a new lncRNA called *TARID*, which arises as an antisense transcript from the Transcription Factor 21 (*TCF21*) genomic locus and is able to activate the transcription of *TCF21* (also known as Capsulin or Pod1 or Epicardin) by inducing TET protein-dependent DNA demethylation [44]. RIP analyses showed a binding between *TARID* and the Growth Arrest and DNA Damage 45 Alpha (GADD45A), which is known to be a regulator of the DNA demethylation process, interacting with TET1 and recruiting DNA repair complexes, which results in the replacement of 5mC with unmethylated cytosine [47,48,49]. Moreover, the sequence of the *TCF21* promoter was captured by biotin-label-*TARID* pulldown, proving that this lncRNA physically interacts with *TCF21* promoter. These experiments suggest that *TARID* might guide GADD45A on the *TCF21* promoter. Even if the authors did not prove the binding of TET proteins to *TARID*, they found that the depletion of TET1/2/3 inhibited *TARID*-mediated demethylation and *TCF21* expression. In addition, the levels of 5hmC were reduced after *TARID* knockdown and increased after ectopic overexpression of *TARID*. Therefore, Arab et al. suggested that *TARID* could bind GADD45A, which in turn recruits TETs together with DNA-repairing proteins on the *TCF21* promoter, inducing demethylation via base excision repair [43].

More recently, Zhou et al. identified a new TET2-interacting lncRNA called *TETILA* by RIP analyses. *TETILA* binds a region from −423 to −438 bp upstream the transcriptional start site (TSS) of the Matrix Metalloproteinase-9 (*MMP-9*) gene, increasing its expression. *TETILA* is highly upregulated in diabetic skin tissues [44]. To model this condition, the authors treated a human keratinocyte cell line (HaCaT) with advanced glycation end-products (AGEs), which are glycated proteins or lipids that are strongly present in an hyperglycemic environment, and demonstrated that the overexpression of *TETILA* promoted TET2 stability and its nuclear translocation [50]. In addition, TET2 activity and 5hmC levels were reduced after the silencing of *TETILA* in AGE-treated cells. RIP analysis revealed that the truncation of two DSBH domains in TET2 protein significantly decreased its binding to *TETILA*, suggesting that they are fundamental for this interaction [50]. Moreover, the same research group reported that *TETILA* knockdown abrogated the occupancy of TET2 on the *MMP-9* promoter and *MMP-9* upregulation [44,50]. In addition, methylated and hydroxymethylated DNA immunoprecipitation (meDIP and hMeDIP, respectively) revealed that high *TETILA* levels promoted 5hmC enrichment on the *MMP-9* promoter region and reduced 5mC levels.

### 2.3. Role of lncRNA in Histone Methylation

Histone methylation is another important regulatory mechanism in transcription regulation. Basic residues (arginine, lysine, and histidine) can be methylated by the addition of methyl (-CH3) groups [51]. This process is catalyzed by a class of enzymes known as histone methyltransferases (HMTs), whereas the reverse process, demethylation, is catalyzed by histone demethylase enzymes (HDMs) [52]. Histone methylation and demethylation regulate gene transcription by modulating the density of chromatin and therefore the accessibility of DNA [52]. lncRNAs have been proposed to bind to specific HMTs and HDMs in order to direct them to their target residues [53].

On another hand, several long non-coding RNAs have been hypothesized to recruit the Polycomb Repressive Complex 2 (PRC2) to specific genomic loci and consequently silence gene expression by promoting histone H3K27-trimethylation [54,55,56].

An Antisense Non-coding RNA in the INK4 locus (*ANRIL*) has been shown to interact with SUZ12, a component of PRC2, and to play a crucial role in regulating SUZ12, binding at INK4B [57,58]. The subsequent recruitment of PRC2 results in H3K27-trimethylation and silencing of INK4B. Indeed, the same study demonstrated that the loss of *ANRIL* or PRC2 causes premature senescence and impaired cellular proliferation in WI38 human fibroblasts [58].

Rinn et al. found that *HOTAIR*, a 2.2 kb non-coding RNA transcribed from the *HOXC* locus, mediates the interaction between PRC2 and histone H3, inducing the H3 lysine 27 trimethylation of the *HOXD* locus, which is repressed in trans *HOXD* transcription [59].

It is noteworthy that histone methylation is associated with both gene repression and activation, as demonstrated by H3K27me3 and H3K4me3.

On that note, Tsai et al. [53] demonstrated that *HOTAIR* is also able to bind the CoREST/REST complex, which includes the demethylase LSD1 and mediates the enzymatic demethylation of H3K4me2, required for the proper repression of *HOX* genes in Drosophila. However, these findings suggest that HOTAIR can act as a modular scaffold, linking to HMTs and HMDs and thereby modulating the pattern of histone modifications on target genes [53].

Another recent study explored the role of the long non-coding RNA nuclear-enriched abundant transcript 1 (*Neat1*) in the methylation of histone 3 lysine 9 dimethylations (H3K9me2) in neuronal cultures. The knockout of *Neat1* in murine neurons resulted in a global reduction in H3K9me2, suggesting a positive correlation between *Neat1* and H3K9me2 [60]. In human cells, *NEAT1* was shown to promote this histone epigenetic mark through two distinct mechanisms: (i) by binding directly to the gene locus of Euchromatic Histone Lysine Methyltransferase 1 (EHMT1) to enhance transcription and (ii) by interacting with both proteins in the EHMT1/2 complex, which are responsible for H3K9me2 [61].

In corroboration with *NEAT1*, the lncRNA metastasis-associated lung adenocarcinoma transcript 1 (*MALAT1*) was also found to be enriched and localized at hundreds of genomic sites with histone methylation marks specifically associated with euchromatin and active transcription [62]. An increase in methylation was noted at H3K4me3 and H3K36me3; however, the molecular mechanisms through which this occurs are unknown.

Similarly, Chen et al. discovered a novel lncRNA termed Low expressed in Bladder Cancer Stem cells (*lnc-LBCS*), able to inhibit bladder cancer progression and chemoresistance. *lnc-LBCS* repress the SRY-box 2 (*SOX2*) transcription, which is essential factor in the self-renewal of bladder cancer stem cell populations [63]. In their study, the authors demonstrated that *lnc-LBCS* binds to the *SOX2* promoter, where it recruits the protein complex consisting of Heterogeneous Nuclear Ribonucleoprotein K (hnRNPK) and Enhancer of Zeste Homolog 2 (EZH2), which then mediates the epigenetic silencing of SOX2 through the formation of H3K27me3 at its promoter [63].

### 2.4. Role of lncRNA in Histone Acetylation

Histone acetyltransferases (HATs) and deacetylases (HDACs) are enzymes that carry out their function across a wide range of organisms, including prokaryotes, plants, fungi, and animals. They play a pivotal role in regulating gene expression and chromatin structure [64,65]. Histone acetylation, mediated by HAT, is primarily associated with promoters and enhancers of actively transcribed genes, whereas it is reduced in repressed genomic regions [66,67]. In contrast, HDACs remove acetyl groups from histones, reducing transcription factor binding to DNA and promoting their degradation. HDACs are divided into two primary groups: the histone deacetylase family (HDAC1–11) and the sirtuin protein family (SIRT1–7). The latter is distinguished by a conserved deacetylase domain and its reliance on specific cofactors [68]. The cross-talk of lncRNAs with histone modifications involves lncRNAs directing or modulating HAT and HDAC activity, thereby influencing chromatin dynamics and gene regulation.

In addition, the interplay between lncRNA, miRNAs, and epigenetic modifications also plays pivotal roles in regulating the gene expression of various biological processes, including gene regulation in cell growth, differentiation, and disease development.

Of particular interest, in 2007, Camblong et al. [69] were the first to demonstrate how the progressive decrease in *PHO84* mRNA levels occurs during aging and involves a heritable histone modification [69]. The long-term stabilization of the *PHO84* antisense appeared to inversely correlate with the expression of *PHO84* mRNA. In *Saccharomyces cerevisiae*, the presence of *PHO84* antisense was found to be destabilized by ribosomal RNA-processing protein 6 (RRP6), a component of the exosome. Yeast cells lacking this protein exhibited stable expression of *PHO84* antisense over time. The progressive decrease in *PHO84* mRNA levels during aging has been shown to be influenced by heritable epigenetic modifications. Histone deacetylation, a common form of epigenetic change associated with transcriptional repression, was observed in this case. Specifically, histone H3 acetylation at lysine 18 decreases over time at the *PHO84* promoter in regions overlapping with the antisense transcript. Furthermore, aged cells lacking HDA1, HDA2, and HDA3 (part of the same histone deacetylase HDA1 complex) do not exhibit a repression of *PHO84* mRNA [69].

Recent studies have re-examined the role of yeast *PHO84* antisense transcripts, revealing that the model of antisense-mediated repression does not consistently apply [70]. The authors found that the sense and antisense transcripts of *PHO84* are positively correlated and provided evidence that the 3′ UTR of *PHO84* functions as a regulatory element for the *PHO84* sense transcript. Furthermore, they proposed that the RNA-binding protein THO1, interacts with the 3′ UTR, facilitating the repression of *PHO84* mRNA through a looping mechanism [70].

The well-characterized *MALAT1* has been shown to impair β cell function by reducing H3 histone acetylation at the *PDX-1* promoter, thereby suppressing PDX-1 expression and insulin secretion, a critical mechanism in the development of type 1 diabetes [71]. An intriguing example of the interplay between lncRNAs and histone acetylation is provided by the discovery that the Antisense Non-coding RNA in the INK4 locus (*ANRIL)* regulates VEGF expression and function in diabetic retinopathy. In this context, *ANRIL* interacts with *p300*, *miR200b*, and EZH2 of the PRC2 complex, inducing a reduction in retinal *p300* levels in diabetic murine models [72]. However, the specific mechanisms may vary depending on the context and cell type [73].

*ANRIL* functions as a molecular scaffold, facilitating the binding of WDR5 and HDAC3 to form WDR5-HDAC3 complexes, which subsequently regulate histone modifications and the transcription of target genes such as *NOX1*. *ANRIL* overexpression could induce cell growth and reactive oxygen species (ROS) production in human aortic smooth muscle cells (HASMCs), a detrimental form of vascular remodeling and disease progression in humans [74].

The Maternally Expressed Gene 3 (*MEG3*) is a long non-coding RNA expressed in a tissue-specific manner and plays a role in the development of several diseases, particularly in cancer. The *MEG3/miR-34a* axis regulates NF-κB deacetylation through SIRT1, and this regulation has a significant impact on the inflammation and apoptosis of retina epithelial cells and on cancer biology in general [75,76]. Indeed, the disruption of this axis can lead to uncontrolled NF-κB activation, which is a hallmark of many types of cancer, such as liver, colorectal, breast, gastric, and pancreatic cancer [76], making it a potential target for therapeutic intervention.

### 2.5. Epigenetic Memory of Histone Acetylation and Chromatin Replication

During each cell division, a human cell will replicate ~2m of DNA within the S-phase time constraints. This process begins at multiple points across the genome, known as replication origins. Among these origins, a special group called “core origins” has been identified. The core origins are shared by various cell types and are responsible for initiating approximately 80% of all DNA replication events in any human cell population [77]. However, the exact positioning of DNA replication initiation sites (origin genomic coordinates) in the human genome remains largely unclear.

Numerous studies have focused on identifying origin sequences by examining their overlap with histone marks [78]. The impact of lncRNAs on epigenetic marks that affect chromatin replication has remained largely unexplored for years, and only recently have new investigations begun to emerge. Histone deposition and their epigenetic marks play a significant role in determining signal for potential replication origins by influencing the accessibility of DNA for the replication machinery, essentially marking areas where replication can initiate. Early studies that highlighted the role of histone acetylation in origin firing established a connection between histone H4 acetylation and replication licensing, particularly through its interaction with replication factors such as the MCM complex via Cdt1 [79,80,81,82,83]. In fact, the acetylation of H3 and H4 is frequently enriched at replication origins, suggesting a relationship between the chromatin state and the locations where replication is initiated [78].

However, DNA replication can only proceed once all proteins and RNA are temporarily dissociated from the double-stranded DNA. After replication, chromatin components are then reassembled behind the replication fork to re-establish the chromatin structure on the newly synthesized DNA [84,85]. Nascent chromatin consists of both parental histones, recycled from disassembled nucleosomes just ahead of the replication fork, and newly synthesized, unmodified histones. In HeLa cells, parental and new histones are incorporated in a mix of 1:1 ratio, though this may vary at specific genomic locations or in different cell types [86]. In this way, histone modifications and chromatin are ensured and allow cells to “remember” their identity and function across cell divisions in a process defined as *epigenetic memory* [84,87].

*HOTAIR*, for instance, has been shown to influence histone acetylation dynamics and to engage chromatin-modifying enzymes [88]. By directing histone acetyltransferases to specific genomic regions, *HOTAIR* could also influence histone acetylation states during DNA replication. However, the molecular mechanism guiding the loading of certain histones and the respective modifications to replication origin firing remains largely unexplored.

Recent findings demonstrated that H2A.Z facilitates the licensing and activation of early replication origins; in particular, its depletion from origin sites induces arrest at the G1/S of Hela cells [89].

In 2021, Ebralidze et al. conducted a pioneering study proposing that the formation and recycling of H2A.Z acetylation are mediated by a specialized class of lncRNAs that are highly expressed in the S phase, termed *SPEARs* (S Phase Early RNAs). These cell cycle-specific lncRNAs, encoded near the promoters of active genes, regulate the acetylated form of the replacement histone H2A.Z and its deposition in the human genome, highlighting their role in chromatin dynamics during replication and transcription [90].

## 3. The Multifaceted Roles of lncRNAs in Epigenetic Regulation and Disease Development

LncRNAs have been implicated in various age-related diseases, dysregulating many cellular processes at the epigenetic level and thus playing roles in disease onset, progression, and prognosis [91,92]. Understanding the functions and regulatory mechanisms of lncRNAs can provide insights into disease mechanisms and potentially lead to the development of novel therapeutic strategies.

**Figure 3:**
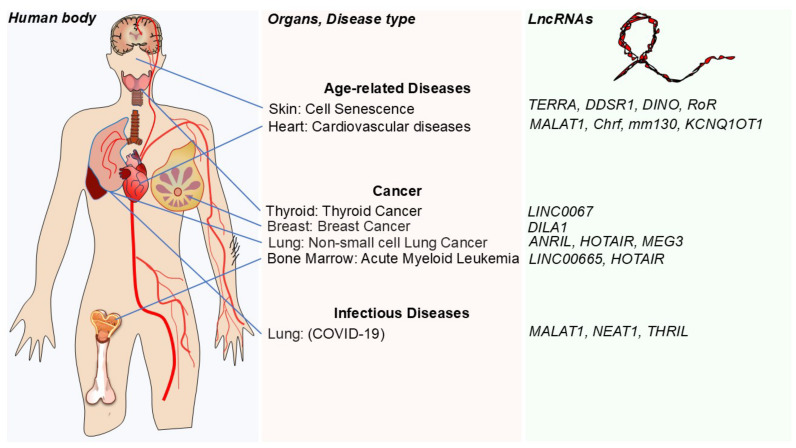
**Diverse roles of lncRNAs in epigenetic regulation and disease progression.** The illustration emphasizes the diverse roles of lncRNAs in shaping epigenetic landscapes throughout the human body. Their impact on chromatin remodeling, DNA methylation, and histone modifications plays a crucial role in various molecular mechanisms driving human diseases. The figure organizes these effects by organ and disease type, including age-related conditions such as cancers as well as complications from infectious diseases.

The following sections explore how lncRNAs modulate epigenetics in the context of common age-related diseases, including cancers and complications arising from infectious diseases, with a summary scheme illustrated in Figure 3.

### 3.1. Role of lncRNAs in Cellular Senescence and Aging: Guardians and Regulators of Genome Stability

Aging is classified as an increasing number of senescent cells that lead to tissue and organ dysfunction. With extending lifespans [93], the prevalence of age-related diseases has risen, and lncRNAs are predicted to play a critical role in disease progression [94,95,96].

Cell senescence is known as a permanent state of dormancy with growth arrest and altered physiological functions [97]. It is initiated by telomere erosion and the exposure of DNA to damaging conditions [98,99]. Telomeres protect chromosomes from degradation, and their length is one of the factors determining aging [98]. They continue to shorten during cell division, and eroded telomeres may fuse and trigger chromosome instability, DNA damage, and apoptosis [100]. Telomeric repeat-containing RNA (*TERRA* lncRNA) regulates deprotected telomere structure and function [101]. *TERRA* lncRNAs interact with SUV39H1, an HMT, to induce methylation at the damaged telomere and aid in the production of heterochromatin [101]. The number of *TERRA* lncRNAs surges with aging due to increased incidents of DNA and telomere damage [101].

The antisense transcript of *TERRA*, known as *ARRET* (Antisense Repeat RNA of *TERRA*), plays a crucial role in the regulation of telomeric function. While *TERRA* is a G-rich RNA transcribed from the telomeric C-rich strand, *ARRET* represents the complementary transcript derived from the G-rich strand [102]. *ARRET* may function as an epigenetic regulator of telomeres by reinforce H3K9me3 deposition at telomeres, promoting a silenced chromatin state by recruiting methyltransferases, e.g., SUV39H1 in mammals and ClLR4 in fission yeast [103,104].

Interestingly, *TERRA* depletion increases telomeric pathologies, including telomere-induced DNA damage foci, with a subsequent loss or duplication of telomeric sequences [105]. Indeed, *TERRA*-induced R-loops (RNA:DNA hybrids) cause DNA damage at telomeric ends and replication stress, particularly in human cancer cells [105].

A recent study that combined RNA-centric epigenomics and proteomics approaches established a map of *TERRA*’s chromatin interaction sites in the mouse genome, identifying *TERRA* and ATRX (Alpha-Thalassemia/Mental Retardation X-Linked), a chromatin remodeler and transcriptional regulator, as sharing hundreds of target genes [106]. The loss or depletion of ATRX also led to pathological consequences, particularly in aging, cancer development, and telomere-associated diseases [107]. In this context, in 2018, Chu et al. demonstrated that ATRX plays the role of a key interactor and antagonist of TERRA competing for telomeric DNA [106].

Other causes of DNA damage leading to cellular senescence include ROS, reactive nitrogen species (NOS), and physical and chemical agents [108]. When DNA is damaged, it goes through a series of repair systems known as the DNA damage response (DDR) [108]. DNA damage-sensitive RNA1 (*DDSR1*) lncRNA takes part in DDR by interacting with the BRCA1/RAP80 complex and sequestering it at the damage site [109]. It also encourages cell survival by inducing homologous recombinational repair by interacting with hnRNPUL1 when the DNA goes through a double-strand break [109]. p53 is known as the master regulator of the cell cycle, and it activates cell cycle arrest and promotes cell senescence when DNA damage is detected [110]. Damage-Induced Non-Coding (*DINO*) lncRNA binds to the C-terminus of p53 and stabilizes its cell cycle arrest activity by colocalizing at the target genes [111]. On the other hand, lncRNA *RoR* interacts with heterogeneous nuclear ribonucleoprotein I (hnRNPI) to induce post-transcriptional repression of p53 [112].

### 3.2. Long Non-Coding RNAs in Age-Related Cardiovascular Diseases: Molecular Regulators of Heart Health and Dysfunction

The incidence of cardiovascular diseases increases with age due to vascular stiffness, altered signaling pathway activation, and decreased cell proliferation [113]. Some characteristics of age-related cardiovascular diseases include atherosclerosis, cardiac hypertrophy, acute myocardial infarction, heart failure, and arrhythmia [113]. Increased levels of lncRNA *MALAT1* activate the WNT/β-Catenin signaling pathway to speed up the endothelial-to-mesenchymal transition, which leads to the dysfunction of human umbilical vein endothelial cells and atherosclerosis progression [114]. In cardiovascular diseases, most lncRNAs operate by binding to ribonucleoproteins (RNPs) and defecting their functions, while few others function by affecting miRNAs and/or DNA binding proteins [115]. An example of this is seen in cardiac hypertrophy, where a decoy lncRNA CH- related factor (*Chrf*) binds to *miR-489,* preventing the degradation of the hypertrophy-related *MYD88* gene and promoting the progression of the disease [116].

In the context of acute myocardial infarction, a guide lncRNA named Wisper (Wisp2 super-enhancer-associated RNA) was identified as a cardiac fibroblast-enriched transcript that plays a regulatory role in cardiac fibrosis following injury.

*Wisper* may interact with specific RNA-binding proteins such as TIA1 (T-cell intracellular antigen 1) and other TIA1 family proteins that regulate RNA splicing, RNA translation, and RNA stress granule formation. In cardiac fibrosis following injury, *Wisper* is associated with TIA1 family proteins controlling the mRNA processing of a profibrotic form of lysyl hydroxylase 2 (*LH2*), which is implicated in collagen cross-linking and stabilization of the extracellular matrix (ECM). *LH2* is a key player in cardiac fibrosis. Its dysregulation contributes to an excessive deposition of collagen and other ECM proteins in heart tissue, leading to stiffening and scarring of the myocardium [117].

An interesting characteristic of age-related heart failure is the increased re-expression of several fetal genes [118]. The lncRNA *mm130* plays a critical role by directly binding to the promoter of *Tbx20* and reactivate fetal gene augmenting programs [118]. Aging also contributes to a deteriorated calcium handling in cardiomyocytes, which gives rise to another cardiovascular-related disease: arrhythmia [119].

Remarkably, the increased expression of the Calcium Voltage-Gated Channel Subunit -Alpha 1C (CACNA1C) is strongly linked to the progression of arrhythmia. Shen et al. demonstrated that the upregulation of the lncRNA *KCNQ1OT1* promotes atrial fibrillation (AF) progression in both AF- and angiotensin II-induced heart (Ang II-induced heart) mouse models. Through their research using starBase (http://starbase.sysu.edu.cn/index.php, accessed on 28 February 2025), Shen et al. confirmed that miR-384 simultaneously binds to both the lncRNA *KCNQ1OT1* and CACNA1C. Furthermore, they demonstrated that an overexpression of *KCNQ1OT1* upregulates CACNA1C by competitively binding miR-38 [120].

## 4. Long Non-Coding RNAs in the Epigenetic of Cancers

### 4.1. Age-Related Factors and lncRNAs in Epigenetic Cell Reprogramming Leading to Malignant Transformation

Cancer is one of the most well-known diseases associated with aging. Long-term exposure to several stress factors, including reactive oxygen species, free radicals, and UV radiation, can cause an accumulation of DNA damage that may lead to cell transformation and eventually to cancer development [121]. The number of senescent cells increases as aging progresses [122]. Although senescent cells are at growth arrest, they remain metabolically active and exhibit the senescence-associated secretory phenotype (SASP) [123]. These cells secrete cytokines, growth factors, and immune modulators that can generate a protumor environment by promoting migration, cell proliferation, and angiogenesis [123]. With aging, many cells demonstrate decreased or altered functions, and the same phenomenon applies to immune cells [124]. The number of innate immune cells is found to be constant with increasing age, but they demonstrate reduced immune functions [125]. On the other hand, adaptive immune cells exhibit a decline in both the number and function of naïve cells, associated with an increase in memory T cells [124]. This decline in immune function prevents older individuals from having an effective tumor immune response [124]. DNA damage and genetic alterations lead to a malfunction of metabolic signaling in cancer cells, and several lncRNAs play important roles throughout the process, either as oncogenes or tumor suppressors [126].

On the same note, long non-coding RNAs have emerged as critical epigenetic regulators of metabolic pathways involved in cancer progression. They influence epigenetic marks in crucial metabolic networks that support tumor growth and survival. Their ability to modulate key pathways such as glucose uptake and adipogenesis, as well as metabolic reprogramming in cancer cells, offers potential avenues for therapeutic intervention [127,128].

*HOTAIR* is an example of a lncRNA associated with glucose uptake. Through its interaction with PRC2, *HOTAIR* induces epigenetic modifications that influence gene expression. This suggests a potential role in regulating genes involved in glucose uptake, ultimately impacting cellular energy homeostasis [129,130,131,132].

Another intriguing candidate in the formation of a link between epigenetics and metabolism is *MALAT1*, whose interaction with EZH2 and involvement in H3K27 trimethylation (H3K27me3) are well-established. This epigenetic mechanism may indirectly regulate genes associated with adaptive oxidative stress and the epigenetic control of mitochondrial metabolism in hepatoma cells, laying the groundwork for further exploration of lncRNAs in tumor metabolic reprogramming [133,134,135].

The following paragraphs highlight both well-established discoveries and the emerging role of lncRNAs as crucial epigenetic modulators in the pathogenesis of various cancer types, including acute myeloid leukemia, non-small-cell lung cancer, breast cancer, and thyroid cancer. This includes their involvement in essential molecular mechanisms that lead to epigenetic changes and drive cancer development.

### 4.2. Role of LncRNAs as Epigenetic Regulators of Acute Myeloid Leukemia (AML)

A 2021 study explored the role of the lncRNA *LINC00665* in 36 AML patients and 36 healthy donors. *LINC00665* expression was upregulated in AML bone marrow cells when compared to the healthy donors [136].

The regulation of the *LINC00665*/*miR-4458*/*DOCK1* axis is crucial in disease progression. In particular, *LINC00665* contains specific binding sites for *miR-4458* [136]. More generally, miRNA sponging by lncRNA can lead to gene activation. This phenomenon occurs when a molecule (often lncRNA or Decoy) is designed to sequester or bind miRNAs, preventing them from interacting with their natural mRNA targets. By “sponging” the miRNA, this approach effectively reduces the miRNA’s repression on target genes, potentially leading to their change on epigenetic marks and/or gene activation. The sponging of *miR-4458* by *LINC00665* leads to the activation of DOCK1 expression, which in turn enhances the GTP-Rac1 complex, a novel player in the promotion of AML tumorigenesis. An increased Rac1 activity promotes tumor proliferation and DNA synthesis, resulting in a poorer prognosis of patients with AML. The simultaneous knock-out of *LINC00665* and *DOCK1* leads to reduced proliferation and progression [136]. This axis could be a potential target for future therapeutic design for treating patients with chemotherapy resistant AML.

Interestingly, in the case of *HOTAIR*, it was shown to activate methylation at the *PTEN* locus by upregulating the expression of DNMT3b, thereby promoting resistance to adriamycin (ADM) in acute myeloid leukemia [137]. The authors first conducted a screening on AML patient bone marrow samples, demonstrating an upregulation of *HOTAIR* and a downregulation of *PTEN*. Functional experiments were then performed to investigate the roles of *HOTAIR* and *PTEN* in ADM resistance, correlating the upregulation of *HOTAIR* and DNMT3b with the downregulation of *PTEN* in both ADM-sensitive and ADM-resistant AML cells. Finally, the methylation of *PTEN* and upregulation of DNMT3b were observed in AML-ADM-resistant cells following *HOTAIR* knockdown. Several molecular mechanisms have been suggested to explain how *HOTAIR* regulates *PTEN* methylation; however, to date, no direct interaction between *HOTAIR* and DNMTs has been demonstrated in AML [137]. A more recent study identified the role of *HOTAIR* in attenuating chronic myelogenous leukemia progression by confirming its direct binding to DNMT1 and inhibiting the methylation of the PTEN gene promoter [138]. Although a similar mechanism may occur in AML, the interaction between HOTAIR and DNMTs has yet to be fully explored, leaving room for further investigation in this area. The lncRNAs that are dysregulated in AML are summarized in Table 2.

### 4.3. LncRNAs Regulate the Epigenetics of Non-Small-Cell Lung Cancer

*CDKN2A* (p16) was the first identified tumor suppressor gene silenced by promoter methylation and associated with lung carcinogenesis [139,140]. In contrast, the methylation status examined at CpG islands of the neighbor gene *CDKN2B* (p15) was found to be nonmethylated for 51 non-small-cell lung cancer patient samples [141].

In this context, numerous studies have demonstrated the oncogenic properties of the long non-coding RNA *CDKN2B-AS1* (*CDKN2B* antisense RNA 1, *ANRIL*) in multiple carcinomas, including thyroid cancer [142], gastric cancer [143], and lung cancer [144]. Additionally, *CDKN2B-AS1* has been found to be overexpressed in NSCLC tissues and serum samples [145]. In cancers other than lung cancer, *ANRIL* has been shown to recruit PRC2 to the *CDKN2A/B* locus, resulting in H3K27me3 modifications and transcriptional repression of tumor suppressor genes [140]. However, its role in shaping the histone methylation profile of *CDKN2B* in NSCLC has not yet been confirmed [146].

c-Myc, a highly studied transcription factor that is also overexpressed in NSCLC, has been shown to bind to the *CDKN2B-AS1* promoter region [147,148]. Yi Lu et al. further confirmed this interaction through chromatin immunoprecipitation assays [148]. c-Myc binding to the *CDKN2B-AS1* promoter induces its transactivation, thereby enhancing A549 cell proliferation [148,149]. However, the precise molecular mechanism underlying *ANRIL*-induced cell proliferation remains not yet fully elucidated.

A widely used example of a lncRNA changing histone marks in the gene regulation of lung cancer models is provided by *HOTAIR* [53,150,151]. *HOTAIR* has been reported to play a critical role in the physical interaction and recruitment of PRC2 and LSD1 (Lysine-Specific Demethylase 1), which are associated with the CoREST (REST co-repressor) complex proteins, to specific genomic regions [53]. PRC2 catalyzes the methylation of histone H3 at lysine 27 (H3K27me3), a modification linked to gene silencing facilitated by the enzymatic action of EZH2 and the structural support of SUZ12, both components of the PRC2 complex. In contrast, the demethylation of H3K4me2/3 is carried out by the enzyme LSD1 (Lysine-Specific Demethylase 1), with *HOTAIR* serving as a modular scaffold that organizes the epigenetic environment to promote its interaction with chromatin [53]. Numerous studies have highlighted *HOTAIR*’s role and its interaction with histone modification complexes, contributing to the epigenetic silencing of tumor suppressor genes. A dysregulation of *HOTAIR* has been associated with tumor progression and metastasis in several cancers [53]. However, how changes in *HOTAIR* expression levels disrupt the delicate balance of PRC2-lncRNA complexes and drive cancer progression, particularly in lung cancer, remains an open question [150].

In 2016, the role of *MEG3* was associated with the epigenetic regulation of the epithelial–mesenchymal transition (EMT) in lung cancer cell line models [152]. More specifically, JARID2 (Jumonji, AT-Rich Interactive Domain 2) has been previously identified as a crucial factor for the proper recruitment of PRC2 to chromatin [153,154,155,156]. Additionally, *MEG3* has been reported to interact with JARID2 in mouse embryonic stem cells (ESCs) [157].

M Terashima et al. first confirmed the interaction of *MEG3* and JARID2 using the RNA immunoprecipitation (RIP) assay. They then examined the correlation between *MEG3* expression levels and EZH2 recruitment at specific genomic loci in A549 and LC-2/ad lung cancer cell lines. The authors proposed an intriguing hypothesis that *MEG3*’s interaction with JARID2 could regulate EZH2 recruitment, thereby facilitating the establishment of H3K27 trimethylation (H3K27me3) in lung cancer cells. This was further supported by chromatin immunoprecipitation (ChIP) assays, which meticulously assessed EZH2 occupancy and H3K27me3 enrichment at regulatory regions of *CDH1* (Cadherin 1, E-cadherin) and *microRNA-200* family genes, including *miR-200b/200a/429* and miR-200c/141 [152]. Dysregulated lncRNAs in non-small-cell lung cancer are reported in Table 3.

### 4.4. LncRNAs in Breast Cancer: Mediators of Tamoxifen Resistance and Disease Advancement

A study conducted in April 2020 explored the role of lncRNAs in breast cancer, highlighting *DILA1* as the key regulator inhibiting the novel oncogene *Cyclin D1*. The study revealed a direct interaction between *DILA1* and *Cyclin D1*, which promotes tamoxifen resistance, a novel endocrine therapy used to treat ER-positive breast cancer, and enhances cell proliferation [158,159]. *DILA1* binds directly to Thr286 of cyclin D1, preventing its phosphorylation and subsequent ubiquitination, which in turn inhibits protein degradation [159]. In both in vitro and in vivo models, Cyclin D1 degradation is increased under *DILA1* knockout conditions, reversing tamoxifen resistance [159].

Another intriguing long non-coding RNA hypothesized to play a role in breast cancer development, particularly through its regulation of HDACs, is *Xist*. *Xist* is a 17 kb long non-coding RNA that, despite being capped, spliced, and polyadenylated, remains retained in the nucleus. Its role has been shown to be essential for X-chromosome inactivation (XCI) during early embryonic development in female mammals [160,161]. *Xist* has been demonstrated to be specialized in recruiting various silencing factors during XCI, including the histone deacetylase 3 (HDAC3), which catalyzes the histone modifications that subsequently induce changes in chromatin conformation [162]

Notably, the removal of H3K27ac is one of the earliest epigenetic events in XCI, leading to chromatin compaction and gene silencing, further reinforcing the transcriptionally repressed state on the inactive X chromosome [163]. In contrast, H3K9ac is an activation mark associated with transcriptionally active euchromatin. In the context of *XIST*-mediated XCI, the removal of H3K9ac is crucial for suppressing gene expression. Initial studies using a combination of immunofluorescence (IF) and XIST RNA FISH techniques attempted to correlate the presence of *Xist* with the absence of H3K9 acetylation in non-cancerous mammary epithelial cells (HMECs) and the breast cancer cell line MDA-MB-436 [159].

Although extensive investigations have focused on understanding the mechanisms underlying XCI, the actual interactions between *Xist* and HDACs remain unexplored in breast cancer [162]. Subsequent studies using mouse ES (embryonic stem) cell line models have demonstrated that the RNA-binding protein SPEN (also known as SHARP, SMRT/HDAC1-associated repressor protein) recruits HDAC3 and plays a role in X-chromosome inactivation by directly interacting with *Xist* [164,165]

These findings could offer new insights into the epigenetic interactions between XIST and HDACs, an area that has remained largely unexplored and lacks sufficient investigation in breast cancer research. Table 4 provides a summary of dysregulated lncRNAs observed in breast cancer, highlighting their potential roles and implications in the disease.

### 4.5. LncRNAs in the Epigenetic Modulation of Molecular Mechanisms in Thyroid Cancer

Thyroid cancer is characterized by uncontrolled cell proliferation and dysregulated cell cycling within the thyroid tissue. This results in excessive cell growth and division, driven by a loss of the normal regulatory mechanisms that govern the cell cycle [169,170]. A thyroid can become overactive and lead to cancer due to various epigenetic factors that regulate key cellular processes, including accelerated metabolism and increased glucose uptake, to optimize their energy supply, which provides a fundamental advantage for growth [171].

A 2021 study investigated the regulation of lactate dehydrogenase (*LDHA*), a novel biomarker for cancer prognosis, and its role in supporting the growth and metastasis of papillary thyroid tumors by mediating glycolysis [172]. It was shown that the lncRNA *LINC0067* has a pivotal role in the STAT3/*LINC0067*/LDHA axis and is responsible for the suppression of cancer progression [172]. The Signal Transducer and Activator of Transcription 3 (STAT3) binds to the promoter of *LINC0067*, activating its transcription, which subsequently regulates the *LDHA* expression. In the context of thyroid cancer, STAT3 exerts a repressive effect on the *LDHA* expression, limiting its involvement with the glycolytic pathway. Indeed, the suppression of LDHA and the resulting decrease in lactate production alter the metabolic flux through glycolysis, which ultimately can prevent tumor cell growth and metastasis [172]. The STAT3/*LINC0067* axis could serve as promising targets for papillary thyroid cancer therapy; however, further research is needed to elucidate the underlying molecular mechanisms by which *LINC0067* suppresses *LDHA* expression. Table 5 presents an overview of the lncRNAs that have been implicated in thyroid cancer, outlining their potential functions and involvement in tumorigenesis.

## 5. LncRNAs in Inflammatory and Infectious Diseases (COVID-19)

LncRNAs were first linked to an infection process in a 2010 study [174]. A unique signature of lncRNA expression was reported in both SARS and influenza infection in in vitro murine studies, suggesting a role of lncRNAs in the regulation of the innate immune response and pathogenesis through the Signal Transducer and Activator of Transcription 1 (STAT1) [174].

Remarkably, a 2020 study examined the transcriptome of primary normal human bronchial epithelial (NHBE) cells and lung biopsies from patients with SARS-CoV, the virus responsible for COVID-19 infection. Comparative analysis between control NHBE cells and SARS-CoV-2-infected NHBE cells revealed that SARS-CoV-2-infected cells displayed 155 upregulated lncRNAs and 195 downregulated lncRNAs compared to the control group [175]. Among the upregulated lncRNAs are *MALAT1* and *NEAT1*, which have been implicated as potential biomarkers in both cancer and human immunodeficiency virus (HIV) [175]. *NEAT1* has previously been identified as a regulatory component of the interleukin 8 (IL-8) signaling pathway, which can activate the antiviral response [176]. In addition, *THRIL*, the TNF-α and Histone-Related Interferon-Inducible Long Non-Coding RNA, has been identified as an essential regulator in the immunoregulatory inflammatory response. *THRIL* plays a crucial role in the induction of TNF-α, which is a key cytokine in the immune response and inflammation. *THRIL* helps mediate the transcription of TNF-α by interacting with p65 (also known as RelA), a subunit of NF-κB (nuclear factor kappa-light-chain-enhancer of activated B cells), a well-known transcription factor complex in the inflammatory signaling pathway [177]. However, it is important to note that many aspects of the molecular mechanisms underlying most of these dysregulated lncRNAs have yet to be fully understood.

Fascinating studies conducted in 2021 introduced a computational approach to reveal the presence of target motifs within the SARS-CoV-2 genome which are capable of specifically binding endogenous human microRNAs and long non-coding RNAs [178]. Interestingly, Natarelli et al. have identified 57 microRNAs containing a “GGG” motif, which facilitates leader sequence recognition, predominantly through offset-6mer sites that enable noncanonical binding of microRNAs to viral RNA. Among the identified lncRNAs, *H19* binds to the 5′UTR of the viral genome, specifically targeting the transcript of the viral gene, which plays a critical role in viral infection [178].

Notably, the concept of leveraging SARS-CoV-2 motifs recognized by selective lncRNAs has highlighted the potential for designing innovative RNA-based antiviral therapeutics. For the same purpose, some lncRNAs identified by Natarelli et al. were proposed as candidate inhibitors of SARS-CoV-2 gene expression and suggested for the use against a range of viral infections, pulmonary arterial hypertension, and related diseases [178].

A comprehensive study conducted in May 2021 investigated differences in lncRNA expression in peripheral blood mononuclear cells (PBMCs) between severe and mild COVID-19 cases, with severity determined by ICU admission status. In the severe infection group of patients (n = 50), increased levels of *LINC02207* and *LINC01127* were observed [179]. In the mild infection group of patients (n = 50), increased levels of *LINC02084*, *LINC02446*, *LINC00861*, *LINC01871*, and *ANKRD44* Antisense RNA 1 (*ANKRD44-AS1*) were observed [179]. However, additional studies are necessary to clarify the specific mechanisms of action and functional roles of these lncRNAs in disease progression.

These findings are consistent with broader research exploring the expression profiles and functions of host lncRNAs, as well as their interaction with miRNAs during SARS-CoV-2 infection. To date, lncRNAs have been studied for their roles in critical processes such as viral invasion, replication, multiorgan dysfunction, and the persistence of symptoms in long COVID. Additionally, structure-specific analyses of lncRNAs have been conducted in order to identify potential biomarkers presenting promising opportunities for addressing both acute COVID-19 and its long-term consequences [180,181,182,183].

## 6. Conclusions

In recent years, lncRNAs have been increasingly recognized as key regulators of epigenetic modifications, including DNA methylation, histone modifications, and chromatin remodeling, which collectively shape gene expression, influence cellular phenotypes, and contribute to various diseases, including cancer. However, unraveling the complex architecture of lncRNA networks remains a significant challenge, yet it is essential for advancing our understanding of cellular regulation. Addressing this complexity requires cutting-edge methodologies in gene and genome scanning designed to systematically characterize the intricate networks of lncRNA interactions with chromatin-modifying enzymes and structural chromatin elements.

The molecular mechanisms by which lncRNAs orchestrate epigenetic marks underscore their critical role in the etiology of diseases, establishing them as a foundational aspect of modern genetic and epigenetic research. Efforts to decode these mechanisms promise not only to deepen our understanding of fundamental biology but also to unlock transformative opportunities for innovative medical interventions.

## Figures and Tables

**Table 1:** Classification of lncRNA by DNMT’s association and activity.

Name of lncRNA	Target Gene	DNMT’s Association and Activity	Reference
*ecCEBPA*	*CEBPA*	*ecCEBPA* halts DNMT1’s catalytic activity and establishes a functional link with *CEBPA* expression.	[23]
*Dali*	*DLGAP5, HMGB2, NOS1*	*Dali* interacts with DNMT1 to regulate transcription at the *POU3F3 locus. DLGAP5, HMGB2,* and *NOS1* display increases in DNA methylation levels after *Dali* knockdown.	[25]
*Dum*	*DPPA2*	Dum is involved in the differentiation of skeletal myoblast by recruiting DNMT1, DNMT3a, and DNMTt3b. In the promoter region, it induces *DPPA2* silencing.	[26]
*Fos* ecRNA	*Fos*	*Fos* ecRNA physically interacts with DNMT1 and DNMT3a, impairing DNA methylation in the promoter region of Fos.	[27]
*DACOR1*	*(CBS*) Cystathionine β-synthase (*SMAD6*), Sma and Mad-related protein 6	*DACOR1* overexpression results in the recruitment of DNMT1 and an increase in DNA methylation in many gene regulatory regions involved in the control of cell metabolism and the TGF-β/BMP signaling pathway.	[28]
*FMR1-AS1*	*FMR1*	*FMR1-AS1* transcription affects the methylation status and expression of *FMR1*. The exact mechanism is still unclear. It is hypothesized to occur by direct interaction with the DNA promoter sequence and/or through DNMT1 inhibition within the *FMR1* promoter region.	[33]

**Table 2:** LncRNAs dysregulated in AML.

Name of lncRNA	Target Gene	Function/Mechanism	Up/Downregulated	Associated Disease	Reference
*LINC00665*	*DOCK1*	*LINC00665/miR-4458*/DOCK1 axis: experimental results indicated that LINC00665 exerted a positive function on AML cells by sponging miR-4458 and that miR-4458 influenced the progression of AML by modulating DOCK1 expression.	Upregulated	AML	[136]
*HOTAIR*	*PTEN*	*HOTAIR* activates methylation at the *PTEN* locus by upregulating the expression of DNMT3b, thereby promoting resistance to adriamycin (ADM) in acute myeloid leukemia.	Upregulated	AML	[137]

**Table 3:** LncRNAs dysregulated in non-small-cell lung cancer.

Name of lncRNA	Target Gene	Function/Mechanism	Up/Downregulated	Associated Disease	Reference
*ANRIL*	Not yet confirmed (NSCLC)	*ANRIL* has been hypothesized to recruit PRC2 to the *CDKN2A/B* locus, resulting in H3K27me3 modifications and transcriptional repression of tumor suppressor genes. Its function in NSCLC has not yet been confirmed.	Upregulated	Non-Small-Cell Lung Cancer	[146]
*HOTAIR*	Not yet confirmed (NSCLC)	*HOTAIR* acts as a bridging scaffold for PRC2 and LSD1/CoREST/REST, needed for histone demethylation (H3K4me2/3) and gene silencing.	Upregulated	Non-Small-Cell Lung Cancer	[150]
*MEG3*	*CDH1* (Cadherin 1, E-cadherin) microRNA-200 family genes	*MEG3’s* interaction with JARID2 regulates EZH2 recruitment, thereby facilitating the establishment of H3K27me3.	*MEG3* is frequently found to be downregulated in NSCLC. It is significantly downregulated in A549 and LC-2/ad (lung adenocarcinoma cell lines).	Non-Small-Cell Lung Cancer	[152]

**Table 4:** LncRNAs dysregulated in breast cancer.

Name of lncRNA	Target Gene	Function/Mechanism	Up/Downregulated	Associated Disease	Reference
*DILA1*	Cyclin 1	*DILA1* binds directly to Thr 286 of cyclin D1 protein, preventing its ubiquitination and subsequent degradation.	Upregulated	Tamoxifen-resistant ER + breast cancer	[158,159,166,167,168]
*XIST*	Not yet confirmed in breast cancer	HDAC3 plays a role in X-chromosome inactivation by directly interacting with *Xist* in the mouse ES cell line.	Upregulated	TNBC	[164,165]

**Table 5:** LncRNAs dysregulated in thyroid cancer.

Name of lncRNA	Target Gene	Function/Mechanism	Up/Downregulated	Associated Disease	Reference
*LINC0067*	*LDHA*	STAT3 binding to the promoter of *LINC0067* activates its expression, which suppresses LDHA.	Downregulated	Papillary Thyroid Cancer	[172,173]

## Data Availability

No new data were created or analyzed in this study. Data sharing is not applicable to this article.
